# RELATIONSHIP BETWEEN CHANGE IN FACE-TO-FACE SOCIAL ACTIVITY AND LONELINESS DURING COVID PANDEMIC IN US ADULTS

**DOI:** 10.1093/geroni/igae098.0347

**Published:** 2024-12-31

**Authors:** Sangha Jeon, Susan Charles

**Affiliations:** University of California Irvine, Irvine, California, United States; University of California Irvine, Irvine, California, United States

## Abstract

**Objectives:**

Participation in diverse face-to-face social activities is linked to low levels of loneliness, but social restrictions during the pandemic were restricted in-person interactions. As a result, many people turned to technology to stay connected to others. The current study examined whether shifts in face-to-face social activity variety correlate with pandemic-related loneliness, adjusting for baseline loneliness and sociodemographic factors. We also examined whether this relationship varies depending on online social activity variety. Method: Using data from the Health and Retirement Study (HRS), we included 1,412 adults aged 50 and older who completed questions about social activity participation in 2016 (Wave1; W1) and 2020 (Wave2; W2).

**Results:**

Regression analyses revealed that those who experienced a greater decrease in face-to-face social activity variety during the pandemic compared to four years prior had higher levels of loneliness compared to those who experienced less change in in their pre-pandemic level of face-to-face social activity variety, after adjusting for demographic, health-related factors and loneliness at W1. In addition, online social activity variety does not moderate the relationship between change in face-to-face social activity variety and later loneliness. Conclusions: Findings suggest that engagement in diverse face-to-face social activities is associated with low level of loneliness, but online social activity variety cannot compensate the loss of opportunities for face-to-face social activities in middle-aged and older adults even in the situation with the restricted availability of face-to-face social interactions.

